# Sustainable approach toward synthesis of green functional carbonaceous 3-D micro/nanostructures from biomass

**DOI:** 10.1186/1556-276X-8-348

**Published:** 2013-08-08

**Authors:** Amirhossein Tavangar, Bo Tan, Krishnan Venkatakrishnan

**Affiliations:** 1 Department of Mechanical and Industrial Engineering, Ryerson University, 350 Victoria Street, Toronto, ON M5B 2K3, Canada; 2 Department of Aerospace Engineering, Ryerson University, 350 Victoria Street, Toronto, ON M5B 2K3, Canada

**Keywords:** Femtosecond laser ablation, Carbonaceous compound, 3-D micro/nanostructures, Rice husk, Wheat straw, Biomass, Green approach

## Abstract

This study proposes a novel technique to synthesize functional carbonaceous three-dimensional (3-D) micro/nanocompounds from agricultural by-products using femtosecond laser irradiation. Biowastes of rice husk and wheat straw are value-engineered to carbonaceous structures in a single-step process under ambient conditions. Our results demonstrate that by controlling the laser fluence, structures with a variety of different morphologies from nanostructures to microstructures can be achieved. Also, the results indicate that altering the laser processing parameters influences the chemical composition of the synthesized structures. This sustainable approach presents an important step towards synthesizing 3-D micro/nanofibrous compounds from biowaste materials. These structures, as-synthesized or as nanocomposite fillers, can have practical uses in electronic, sensing, biological, and environmental applications.

## Background

Functional carbonaceous micro/nanostructures have drawn considerable attention in the past few years and are considered one of the most promising materials of the human future life [1]. They have been broadly used in technological applications in different areas such as nanoelectronics, efficient energy storage, catalysis, sustainable chemical technology, and biomedical and environmental sciences [1,2]. Functional nanostructured carbon materials have been prepared in a wide range of morphologies and structures either in form of different carbon allotropes or in complex compound structures, e.g., carbon nanotubes [3], nanospheres [4], nanodiamond [5], carbon nanofibers [6], and carbon-based hybrid nanostructures [7-10]. Thus far, several fabrication approaches such as hydrothermal carbonization [11], carbonization [12], and arc discharge [13] have been reported for the preparation of carbonaceous nanostructures. A special interest has been directed toward approaches that synthesize carbonaceous micro/nanostructures from renewable resources not only with regards to the economic point of view but also with respect to their sustainability and green, nontoxic routes.

Biomass, particularly agricultural by-products, is an abundant low-cost carbon source that can be processed to synthesize functional carbonaceous materials. Rice husk and wheat straw are lignocellulosic materials containing high-concentrated carbon. They possess several potential advantages such as low price, copious renewable source, biodegradability, and high specific strength and stiffness [14].

Although numerous studies have reported the synthesis of carbonaceous nanomaterials from pure xylose, glucose, cyclodextrin, sucrose, starch, etc., only few researches have been conducted to produce carbonaceous micro/nanostructures from natural resources [15]. Most of the previous studies employed hydrothermal carbonization process, which requires catalysts and high temperatures and pressures [15]. Therefore, a sustainable green approach to synthesize carbonaceous nano/microstructures from biomass in a single-step process under ambient conditions would be of great interest.

In this work, we have proposed a novel technique to engineer carbonaceous nano/microstructures from rice husks and wheat straws using femtosecond laser processing. To the best of the authors' knowledge, this is the first time that 3-D nano/microstructures have been synthesized from rice husks and wheat straws using laser ablation. The laser pulses hit rice husk and wheat straw powders and generate a mass quantity of nanoparticles, leading to interwoven micro/nanostructures after further nucleation and collision. The morphology of the structures has been studied using scanning electron microscopy (SEM). The chemical composition of the structures has been analyzed using energy-dispersive X-ray spectroscopy (EDS) analysis.

## Methods

**Figure 1:**
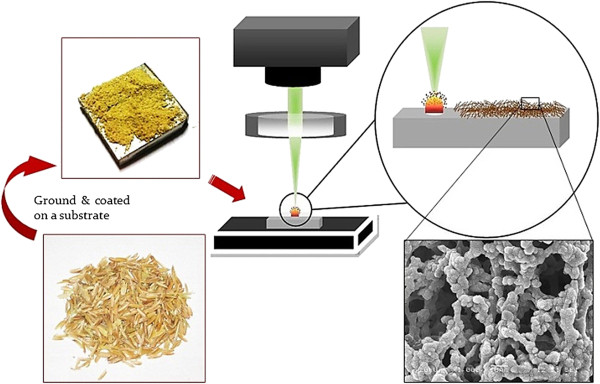
Experimental procedure.

Rice husks and wheat straws were washed with distilled water and dried overnight in an incubator at 50°C. They were then ground into powder and coated on Si substrates. The specimens were irradiated by single-point femtosecond laser processing at different laser dwell times under ambient conditions. Altering the laser dwell time, the time that the laser beam irradiates a particular point on the substrate, allows controlling the number of pulses used to perform laser point processing. The laser source utilized was a 1,040-nm wavelength direct diode-pumped Yb-doped fiber amplified ultrafast laser system. The laser pulse repetition rate ranged from 200 kHz to 26 MHz. The maximum output power of the laser and the laser pulse width were 15.5 W and 214 fs, respectively. This system operates under low-noise performance due to the solid state operation and high spatial mode quality of fiber lasers. Also, all the laser parameters, such as laser repetition rate, pulse width, and beam power, were computer-monitored, which allowed a precise interaction with the performed experiments. The schematic diagram of the synthesis procedure is depicted in Figure 1. The morphology and chemical composition of the micro/nanostructures were characterized using SEM and EDS analyses, respectively.

## Results and discussion

**Figure 2:**
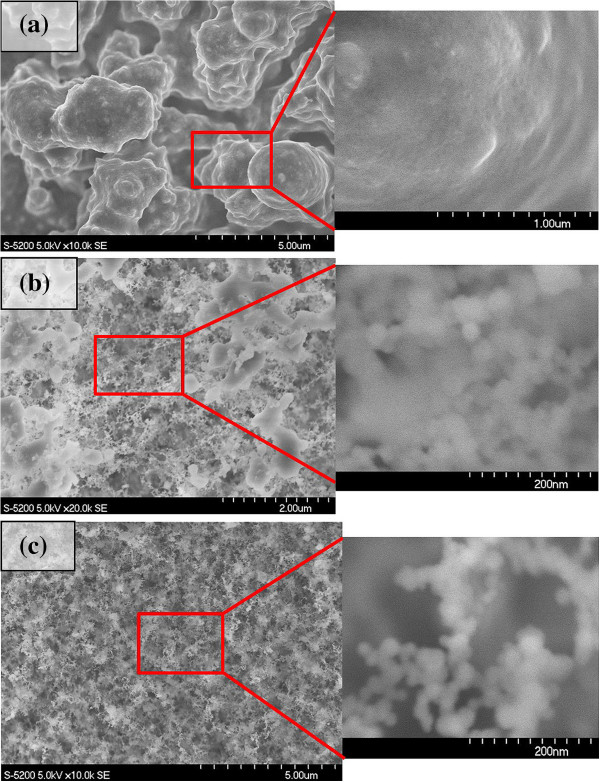
**SEM micrographs of the structures synthesized from rice husks by 2,600 consecutive laser pulses.** The laser pulse energies were **(a)** 0.19, **(b)** 0.38, and **(c)** 0.58 mJ.

The morphology and chemical composition of the synthesized structures are influenced by various laser parameters. First, we investigated the effect of pulse energy on the porosity and size of the structures. Figure 2 shows the SEM images of the structure synthesized by ablating rice husk substrates by 2,600 consecutive laser pulses with different pulse energies. A closeup view of the structures produced by pulses with energy of 58 mJ, shown in Figure 2a, shows that they are comprised of self-assembled closed rings and bridges in which nanoparticles are aggregated together. Figure 2b,c depicts the structures synthesized by the same number of pulses but at different pulse energies.

Given a constant number of pulses, the laser fluence is reduced by decreasing the laser pulse energy. The laser-induced structures are results of particle aggregation. Particle aggregation takes place as part of vapor condensation by the collision of nucleus. To generate nanofibrous structures, an immense amount of nanoparticle aggregation is required. Therefore, continuous arrival of the laser pulses is needed in order to ablate the target material great enough to maintain the plume nucleus density at the critical level. Hence, critical amount of laser fluence should be transferred to the substrate in order to initiate the plume and keep it at the certain level. As a result, the formation of nanofibrous structures is not possible in lower laser pulse energies, and instead, microstructures would be generated.

The evaporation rate by a single laser pulse ablation is a function of material properties and laser parameters [16]:

(1) $$\langle R_{\mathrm{evp}}\rangle {}_{\mathrm{therm}}\propto {\left(\frac{P_{\mathrm{avg}}}{R_{\mathrm{rep}}A_{\mathrm{foc}}}\right)}^{1/2}={\left(\frac{P_{\mathrm{pulse}}}{A_{\mathrm{foc}}}\right)}^{1/2}\left[\frac{\mathrm{atoms}}{{\mathrm{cm}}^{2}}\right],$$

Here, *P*_avg_ is the average power (in *W*), measured directly from incident laser pulse, *R*_rep_ (in s^−1^) is the laser pulse repetition rate, *P*_pulse_ = *P*_avg_/*R*_rep_ is the laser pulse energy, and *A*_foc_ (in cm^2^) is the irradiation focal spot area. It can be obtained by calculating the theoretical laser minimum spot diameter (*D*_0_) as $D_{0}\approx \frac{1.27\lambda_{0}f}{D},$ where *λ*_0_ is the wavelength of the laser, *f* is the effective focal length of the lens, and *D* denotes the laser beam diameter.

As Equation 1 suggests, increasing the laser average power results in a rise in the total laser energy flux transferred to the irradiated spot. The higher transferred laser energy flux for the optimum evaporation regime leads to an increase in the number of evaporated particles; then, the deposition rate of synthesized structures will be analogous to the number of evaporated particles.

**Figure 3:**
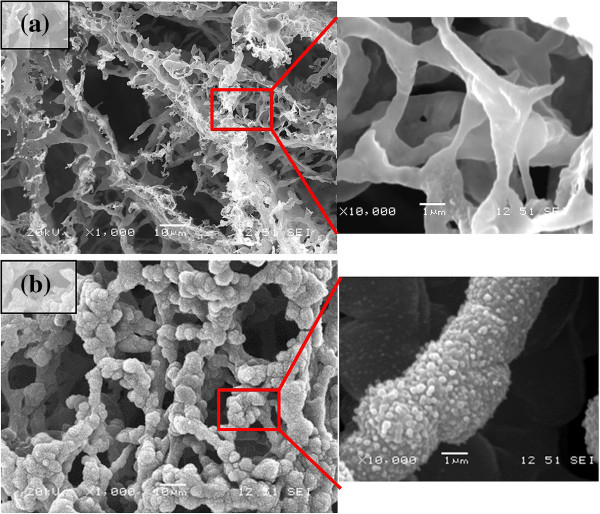
**SEM micrographs of the structures synthesized from rice husks by 1,300 consecutive laser pulses.** The laser pulse energies were **(a)** 0.19 and **(b)** 0.38 mJ.

**Figure 4:**
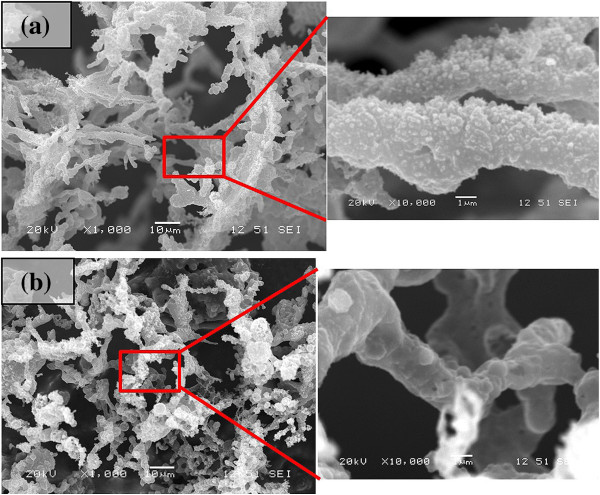
**SEM micrographs of the structures synthesized from wheat straws by 1,300 consecutive laser pulses.** The laser pulse energies were **(a)** 0.19 and **(b)** 0.38 mJ.

The experiments were carried on at different numbers of laser pulses on both rice husk and wheat straw specimens. Figures 3 and 4 illustrate the structures synthesized at different numbers of laser pulses on rice husk and wheat straw substrates, respectively. Decreasing the number of pulses hitting the target leads to a reduction in the laser fluence transferred to a substrate. This results in a decrease in plume volume and nucleus density inside it, which will lead to the generation of microstructures rather than nanofibrous structures.

**Figure 5:**
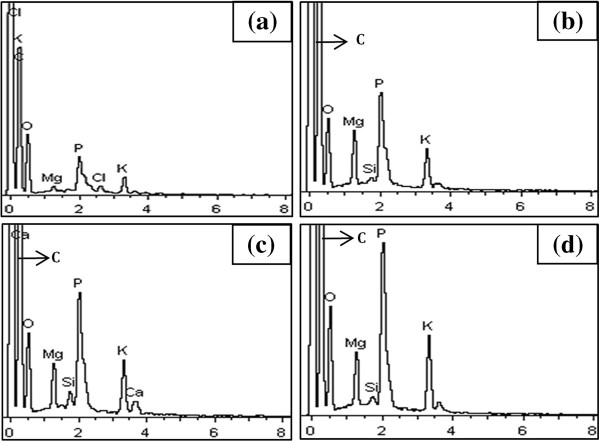
**EDS analyses of unprocessed rice husks and synthesized structures. (a)** Unprocessed rice husks and structures generated from rice husks by 2,600 consecutive laser pulses with pulse energies of **(b)** 0.19, **(c)** 0.38, and **(d)** 0.58 mJ.

**Figure 6:**
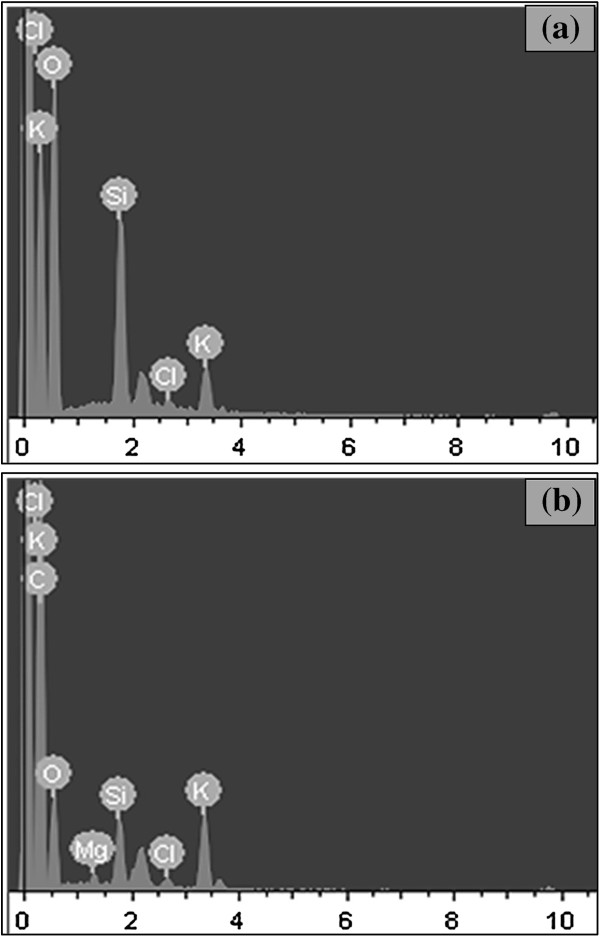
**EDS analyses of unprocessed wheat straws and synthesized structures. (a)** Unprocessed wheat straws and **(b)** structures synthesized from wheat straws by 2,600 consecutive laser pulses with pulse energy of 0.19 mJ.

EDS analyses in Figures 5 and 6 compare the composition changes of the structures synthesized by 2,600 consecutive laser pulses at pulse energies of 0.19, 0.38, and 0.58 mJ on rice husk and at pulse energy of 0.19 mJ on wheat straw, respectively. Since the experiments have been carried out at ambient conditions, the presence of oxygen is noticeable in the EDS graphs. For the unprocessed rice husk and structures synthesized at lower laser pulse energies, silicon and other inorganic elements are not detectable or barely detectable due to a high amount of organic compounds on the substrates. On the other hand, at higher laser pulse energies, the organic part might be burned away partially, so the other inorganic elements could be distinguished. Comparing the unprocessed and the processed structures, one can note that elements, such as chlorine, which are not in favor, has been removed for rice husk samples after laser ablation.

An increase in the number of pulses arriving at the same spot on the substrate results in a rise in the total laser energy flux transmitted to the spot. The higher transmitted laser energy flux for the optimum evaporation regime causes an increase in the number of evaporated particles, which in return will lead to a higher amount of deposited structures. The number of atoms evaporated from the same spot by successive pulses reads [16]:

(2) $$N_{\mathrm{MP}}=N_{P}N_{\mathrm{pulse}}=R_{\mathrm{evp}}A_{\mathrm{foc}}N_{\mathrm{pulse}}\left[\mathrm{atoms}\right],$$

where *N*_p_ is the number of evaporated particles per single pulse [16]:

(3) $$N_{P}=R_{\mathrm{evp}}A_{\mathrm{foc}}\left[\mathrm{atoms}\right].$$

Here, *N*_pulse_ is the number of consecutive pulses hitting the target, and *R*_evp_ is evaporation rate. After irradiation, plume temperature and pressure start to decrease leading to condensation and nucleation. The great amount of nuclei leads to the growth of particles, which will aggregate into interwoven structures after further collision. Since the rate of deposition of generated structures is proportional to the number of evaporated particles, denser structures are synthesized when specimens are targeted by higher energy laser pulses. This is in agreement with our experimental results where denser micro/nanostructures were observed when the targets were processed at higher energy pulses.

The proposed method suggests considerable promise for the synthesis of 3-D micro/nanostructures from green materials to develop new functional compound materials for various applications.

## Conclusions

This work presented a laser-based approach to synthesize carbonaceous micro/nanofibrous structures from rice husks and wheat straws. To the best of our knowledge, this is the first time that synthesizing 3-D micro/nanofibrous structures generated from rice husks and wheat straws using femtosecond laser have been reported. The morphological analyses by SEM confirmed that fabricated structures were composed of approximately uniform 3-D structure at micro and nano sizes. Further experiments showed that by altering the laser pulse energy and the number of laser pulses, different structures from micro- to nanoarchitectures with different porosities and features could be achieved. The EDS analyses confirmed that laser irradiation affected the chemical composition as well; part of the organic matter is believed to be burned away owing to the laser irradiation. This approach suggests a promising step towards engineering green 3-D platforms from sustainable materials. The as-engineered carbonaceous materials would have very broad practical applications in a variety of areas, such as environmental, catalytic, electronic, sensing, and biological applications. They can also be utilized to form biodegradable nanocomposites with other materials, e.g., polymers.

## Competing interests

The authors declare that they have no competing interests.

## Authors' contributions

AT and KV conceived and designed the experimental strategy. AT prepared the specimens, performed the experiments, and wrote the manuscript. BT and KV helped with the editing of the paper. All authors read and approved the final manuscript.
